# The Correlations and Predictive Capabilities of the “Life's Essential 8” With Respect to All‐Cause Mortality and Cardiovascular Disease Mortality Risks in Individuals Experiencing Sleep Disorders: A Prospective Cohort Study From the NHANES (2005–2014)

**DOI:** 10.1002/clc.70336

**Published:** 2026-04-27

**Authors:** Jia Wei, Tengfei Ji, Yide Yuan, Su Liu, Qing Yan, YuYang Zhao, Lan Yang, Jiahong Xue

**Affiliations:** ^1^ Department of Cardiology The Second Affiliated Hospital of Xi'an Jiaotong University Xi'an Shaanxi China; ^2^ Department of Oncology and Cardiology Xinjiang Medical University Affiliated Cancer Hospital Urumqi Xinjiang China

## Abstract

**Background:**

Life's Essential 8 (LE8) is a framework for assessing cardiovascular health (CVH). Individuals with sleep disorders face an elevated risk of cardiovascular disease (CVD). This study aims to investigate the prognostic value of the LE8 score in predicting mortality among individuals with sleep disorders.

**Methods:**

The prospective cohort study included 1606 adults (aged ≥ 20 years) diagnosed with sleep disorders from the National Health and Nutrition Examination Survey (NHANES) 2005–2014. LE8 scores were categorized into three groups: Low CVH (0–49), Moderate CVH (50–79), and High CVH (80–100). Kaplan‐Meier survival curves were used to compare mortality across these groups. Weighted multivariable Cox proportional hazards models were employed to investigate the relationship between LE8 scores with all‐cause and CVD mortality. The Boruta algorithm was applied for feature selection, and six machine learning (ML) algorithms were utilized to predict all‐cause mortality.

**Results:**

During a median follow‐up of 103 months, 282 deaths occurred, including 66 CVD‐related deaths. The weighted multivariable Cox models revealed that higher LE8 scores were significantly associated with a lower risk for both all‐cause mortality (HR = 0.85, 95% CI, 0.73–0.99) and CVD mortality (HR = 0.72, 95% CI, 0.56–0.93). Among the evaluated ML algorithms, the Gradient Boosting Decision Tree (GBDT) model exhibited the highest area under the curve (AUC) for predicting all‐cause mortality.

**Conclusion:**

Higher LE8 scores are independently associated with a decreased risk of all‐cause and CVD mortality among patients with sleep disorders. These findings highlight the importance of optimizing overall CVH in the clinical management of sleep disorders.

## Graphical abstract

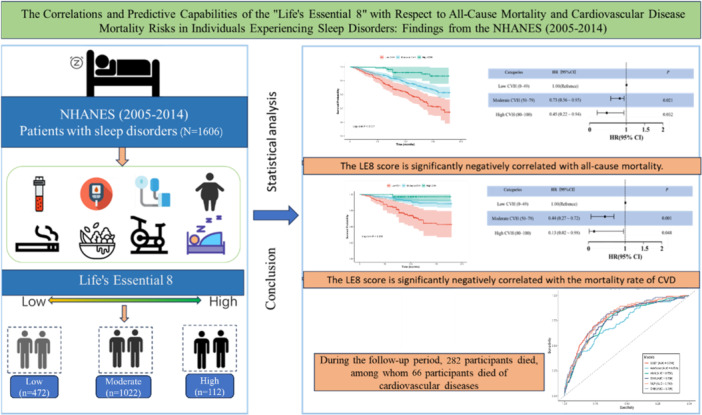

## 1 Introduction

Sleep disorders, encompassing conditions such as insomnia, obstructive sleep apnea syndrome, and circadian rhythm disturbances, exhibit a notably high global prevalence [1]. Epidemiological data from industrialized nations suggest that approximately 25% to 33% of the population experience varying degrees of sleep‐related issues, with roughly 10% suffering from chronic insomnia [2]. These disorders are significantly associated with the development of various chronic diseases and are recognized as leading contributors to sudden cardiac death and traffic accidents [3, 4, 5, 6]. In the United States, the direct and indirect societal costs associated with sleep disorders surpass $100 billion annually, underscoring their status as a critical public health concern [7]. Current research provides compelling evidence linking sleep disorders to increased all‐cause and cardiovascular disease (CVD) mortality within the general population. The underlying mechanisms likely include chronic inflammation, neuroendocrine dysregulation, and metabolic dysfunction [8]. Furthermore, individuals suffering from sleep disorders frequently present with a cluster of modifiable risk factors, such as suboptimal dietary habits, insufficient physical activity, and inadequate blood glucose regulation, which may collectively intensify adverse health outcomes [9, 10, 11].

Life's Essential 8 (LE8) represents a comprehensive framework established by the American Heart Association (AHA) for the assessment of cardiovascular health (CVH) [12]. This framework encompasses two primary dimensions: health behaviors (dietary habits, physical activity, nicotine exposure, and sleep health) and health factors (body mass index [BMI], blood lipids, blood glucose, and blood pressure). These eight components are intricately interrelated and collectively exert a profound influence on the cardiovascular system. Adherence to the healthy lifestyle metrics promoted by LE8 has been demonstrated to substantially reduce the risk of CVD and all‐cause mortality within the general population [13]. Empirical evidence suggests that lower LE8 scores correlate with an increased prevalence of various conditions, including obstructive sleep apnea syndrome, congestive heart failure, and chronic kidney disease [14, 15, 16]. Furthermore, LE8 scores exhibit a strong inverse correlation with adverse outcomes in individuals with hypertension, heart failure, atrial fibrillation, non‐alcoholic fatty liver disease, and cancer [17, 18, 19, 20, 21].

While LE8 has demonstrated robust predictive validity for cardiovascular outcomes across diverse populations, its applicability in high‐risk subgroups, particularly those affected by sleep disorders, remains inadequately examined. Sleep disorders, acting as a unique physiological stressor within the LE8 framework, may attenuate the beneficial impacts of other health determinants or interact synergistically with them, thereby influencing disease risk and mortality. Considering the substantial prevalence of sleep disorders and the immense socioeconomic burden associated with CVD, it is imperative to investigate the prognostic significance of LE8 in this specific population.

**Figure 1:**
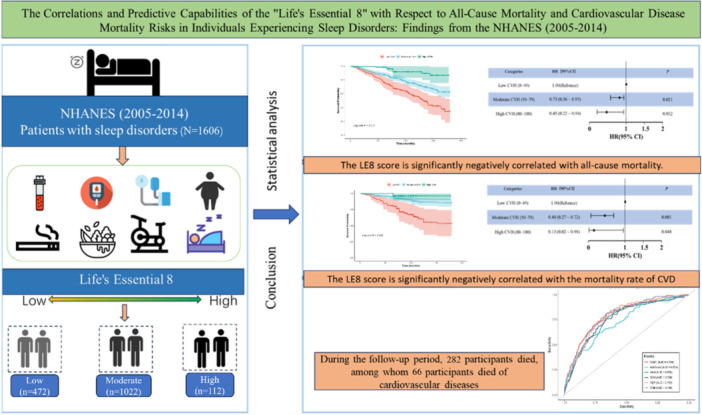
Graphical abstract summarizing the study design and primary findings. The study shows the inverse correlation between Life's Essential 8 (LE8) scores and the risks of all‐cause and cardiovascular disease (CVD) mortality among patients with sleep disorders using data from NHANES 2005–2014.

Utilizing data from the NHANES (2005–2014), this study aims to: (1) quantify the association between LE8 scores and the risk of all‐cause and CVD mortality in adults with sleep disorders, and (2) assess the ability of LE8‐integrated machine learning (ML) models to predict adverse outcomes. The ultimate goal is to provide scientific evidence and practical guidance for health management and CVD prevention in patients experiencing sleep disorders (Figure 1).

## 2 Methods

### 2.1 Study Population

NHANES is a continuous series of cross‐sectional surveys conducted by the National Center for Health Statistics (NCHS) to evaluate the health and nutritional status of adults and children in the United States. The survey employs a stratified, multi‐stage probability sampling design to ensure the cohort accurately reflects the non‐institutionalized civilian population. The NHANES protocol was approved by the NCHS Institutional Review Board, and written informed consent was obtained from all participants.

**Figure 2:**
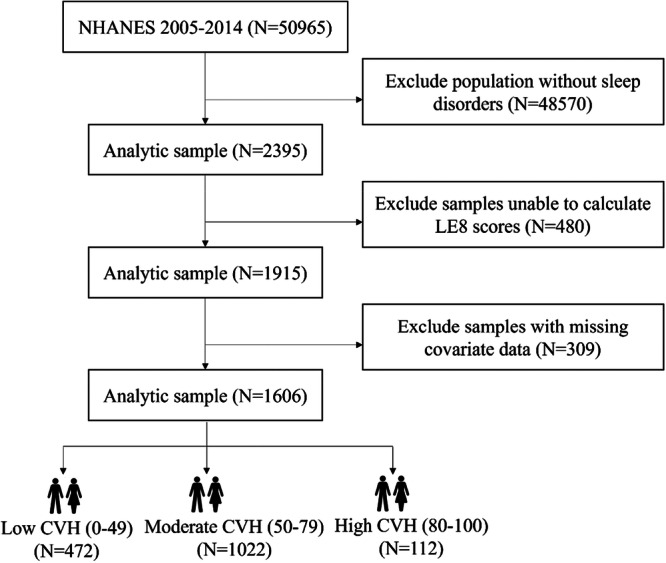
Flowchart detailing the selection process of the study population.

A total of 2395 adults (aged ≥ 20 years) with sleep disorders were identified from the NHANES dataset spanning the years 2005 to 2014. Exclusion criteria (Figure 2) were applied to remove participants missing LE8 scores (*n* = 480) or incomplete covariate data (*n* = 309). The final analytical sample comprised 1606 participants. This study adheres to the Strengthening the Reporting of Observational Studies in Epidemiology (STROBE) reporting guidelines [22].

### 2.2 Definition of Life's Essential 8

The AHA defines the LE8 score as a metric for assessing CVH [12]. This score encompasses four health behaviors (diet, physical activity, nicotine exposure, and sleep health) and four health factors (BMI, blood lipids, blood glucose, and blood pressure). Each of the eight indicators is assigned a score ranging from 0 to 100, and the overall LE8 score is calculated as the unweighted average of these individual components. Consistent with AHA guidelines, LE8 scores were classified into three CVH categories: Low CVH (0–49), Moderate CVH (50–79), and High CVH (80–100). The specific algorithms used to calculate LE8 components using NHANES data were detailed in Table S1.

### 2.3 Definition of Sleep Disorders

Sleep disorders were defined based on the self‐reported physician diagnosis questionnaire. Participants who answered “Yes” to the question *Have you ever been told by a doctor or other health professional that you have a sleep disorder?* were categorized as having sleep disorder. It is noteworthy that during the 2005–2006 and 2007–2008 survey cycles, NHANES collected specific information on the type of diagnosed sleep disorder. The reported subtypes predominantly included sleep apnea, insomnia, restless legs syndrome, and other unspecified sleep disturbances. However, this detailed subtype questionnaire was discontinued in subsequent survey cycles (2009–2014). Consequently, to maintain cohort consistency and maximize statistical power across the entire decade of data, our primary analysis utilized the overarching “sleep disorder” classification rather than stratifying by individual subtypes.

### 2.4 Definition of Mortality

Mortality status and cause of death were ascertained through December 31, 2019, by linking NHANES data to the National Death Index (NDI). The International Classification of Diseases Tenth Revision (ICD‐10) was utilized to specify the cause of death. CVD‐related mortality was defined as deaths resulting from heart diseases or cerebrovascular diseases (ICD‐10 codes I00‐I09, I11, I13, I20‐I51, I60‐I69).

### 2.5 Research Variables

To minimize confounding bias while avoiding over‐adjustment, covariates were carefully selected and categorized as follows: demographic factors included age, gender, and race (categorized as Mexican American, Non‐Hispanic White, Non‐Hispanic Black, Other Hispanic, and Other Race); socioeconomic factors included marital status (classified as married/cohabiting, divorced/widowed/separated, or never married), education level (categorized as less than high school, high school, or college or above), and poverty‐to‐income ratio (categorized as low [< 1.3], middle [1.3–3.4], or high [≥ 3.5]); lifestyle factors included alcohol consumption, which was classified as not at all, medium, or high (drink alcohol more than 4 days per week); medical condition included baseline CVD status and depressive state; and baseline antidiabetic medication use. Depression was defined using the Patient Health Questionnaire‐9 (PHQ‐9), with a score of ≥ 10 indicating clinically significant depressive symptoms [23].

### 2.6 Statistical Analysis

Because NHANES utilizes a complex sampling design, appropriate survey weights (“WTMEC2YR”), as provided by NHANES, is utilized to assign a weight to each participant. The sum of these weights allows the raw sample to be extrapolated to represent the total U.S. civilian non‐institutionalized population. For the baseline characteristics, continuous variables were reported as means with standard deviations (SD), and categorical variables were reported as unweighted counts with percentages.

To ensure the statistical validity of our models and preclude the risk of over‐adjustment, we assessed multicollinearity by calculating the variance inflation factor (VIF), with a VIF value exceeding 5 indicating a significant multicollinearity.

Kaplan‐Meier (KM) survival curves were generated to illustrate all‐cause and CVD mortality rates across the three LE8 categories, with differences assessed via log‐rank test. Weighted Cox proportional hazards models were employed to examine the association between the LE8 scores and mortality, with hazard ratios (HRs) and 95% confidence intervals (CIs) calculated. LE8 was evaluated both as a continuous variable (per 10‐point increment) and as a categorical variable. Three models were constructed: Model 1 which was unadjusted; Model 2 adjusted for age, gender, race, education, poverty, and marital status; and Model 3 which was fully adjusted, adding alcohol consumption, baseline CVD status, depressive state and medication use.

Restricted cubic spline (RCS) analysis, adjusted for the covariates in Model 3, was performed to explore potential non‐linear dose‐response relationships between LE8 scores and mortality risk. Subgroup analyses were conducted by stratifying participants according to age, gender, race, BMI, baseline CVD status and depressive state, with interaction terms tested to evaluate effect modifications.

We established predictive models using six ML algorithms: Adaptive Boosting (AdaBoost), Gradient Boosting Decision Tree (GBDT), Multi‐Layer Perceptron (MLP), Support Vector Machine (SVM), K‐Nearest Neighbors (KNN), and Gaussian Naive Bayes (GNB). These specific algorithms were deliberately selected to encompass a diverse array of learning paradigms, thereby ensuring a comprehensive methodological evaluation. Specifically, ensemble tree‐based methods (GBDT and AdaBoost) are highly robust against outliers and excel at capturing complex, non‐linear interactions among clinical variables. SVM and MLP offer powerful high‐dimensional mapping and neural network capabilities, whereas KNN and GNB serve as reliable distance‐based and probabilistic baselines, respectively. The Boruta algorithm was employed for feature selection to identify variables with significant predictive importance (Z‐scores higher than shadow features). To rigorously validate the models and mitigate the risk of overfitting, the dataset was randomly partitioned into a training set (60%) and a hold‐out testing set (40%). During the model training phase, a fivefold cross validation strategy was implemented within the training set to optimize hyperparameters. The fully tuned models were subsequently evaluated blindly on the unseen testing set. Model performance was evaluated using the Area Under the Receiver Operating Characteristic curve (AUC). Calibration curves and Decision Curve Analysis (DCA) were generated to assess predictive accuracy and clinical utility, respectively.

Finally, sensitivity analyses were performed to confirm the robustness of the primary findings. First, participants who died within the first 2 years of follow‐up were excluded to minimize potential reverse causation, where undiagnosed severe illnesses might concurrently lower LE8 scores and cause early death. Second, the association of LE8 levels with mortality were assessed according to tertiles of LE8 scores, to ensure our results were not artifacts of the AHA‐defined categorical cutoffs. Third, to address potential attrition bias from missing data, the Cox regression analyses were performed with missing data addressed using multiple imputation by chained equations (MICE).

All statistical analyses were conducted using R software (version 4.2.2). A two‐sided *p* value < 0.05 was considered statistically significant.

## 3 Results

### 3.1 Baseline Characteristics

This study included a cohort of 1606 adults diagnosed with sleep disorders. By applying the appropriate NHANES survey weights (“WTMEC2YR”) to account for the complex, multi‐stage probability sampling design, this sample mathematically extrapolates to a nationally representative population of 13,678,416 non‐institutionalized adult residents in the US. Table 1 detailed the baseline characteristics stratified by LE8 CVH categories. Individuals with higher LE8 scores were generally younger, and were predominantly female, non‐Hispanic White, highly educated and with higher incomes. In contrast, participants with Low CVH scores (0–49) carried a significantly higher burden of comorbidities (*p* < 0.05).

**Table 1:** Baseline characteristics of the study sample stratified by LE8 scores.

Characteristics	Overall	Low CVH (0–49)	Moderate CVH (50–79)	High CVH (80–100)	*p*
Total	1606	472	1022	112	
Age (mean ± SD)	53.16 (14.99)	54.27 (12.96)	53.23 (15.53)	47.87 (16.86)	< 0.001
Gender (*n*, %)					0.003
Male	862 (53.7)	246 (52.1)	572 (56.0)	44 (39.3)	
Female	744 (46.3)	226 (47.9)	450 (44.0)	68 (60.7)	
Race (*n*, %)					< 0.001
Mexican American	147 (9.2)	37 (7.8)	99 (9.7)	11 (9.8)	
Non‐Hispanic Black	344 (21.4)	136 (28.8)	196 (19.2)	12 (10.7)	
Non‐Hispanic White	888 (55.3)	234 (49.6)	579 (56.7)	75 (67.0)	
Other Hispanic	143 (8.9)	43 (9.1)	95 (9.3)	5 (4.5)	
Other Race	84 (5.2)	22 (4.7)	53 (5.2)	9 (8.0)	
Education (*n*, %)					< 0.001
Below high school	333 (20.7)	143 (30.3)	178 (17.4)	12 (10.7)	
High school	381 (23.7)	126 (26.7)	244 (23.9)	11 (9.8)	
College or above	892 (55.5)	203 (43.0)	600 (58.7)	89 (79.5)	
Marital status (*n*, %)					0.001
Divorced/widowed/separated	968 (60.3)	262 (55.5)	644 (63.0)	62 (55.4)	
Married/cohabiting	202 (12.6)	62 (13.1)	113 (11.1)	27 (24.1)	
Never married	436 (27.1)	148 (31.4)	265 (25.9)	23 (20.5)	
Poverty ratio (*n*, %)					< 0.001
Low	1034 (64.4)	372 (78.8)	619 (60.6)	43 (38.4)	
Medium	288 (17.9)	54 (11.4)	210 (20.5)	24 (21.4)	
High	284 (17.7)	46 (9.7)	193 (18.9)	45 (40.2)	
BMI (mean ± SD)	33.27 (8.42)	38.19 (9.07)	31.81 (7.21)	25.85 (4.32)	< 0.001
Alcohol consumption (*n*, %)					< 0.001
Not at all	491 (30.6)	174 (36.9)	299 (29.3)	18 (16.1)	
Medium	1111 (69.2)	296 (62.7)	721 (70.5)	94 (83.9)	
High	4 (0.2)	2 (0.4)	2 (0.2)	0 (0.0)	
CVD status (*n*, %)					< 0.001
No	1212 (75.5)	294 (62.3)	817 (79.9)	101 (90.2)	
Yes	394 (24.5)	178 (37.7)	205 (20.1)	11 (9.8)	
Depressive state (*n*, %)					< 0.001
No	1187 (73.9)	285 (60.4)	803 (78.6)	99 (88.4)	
Yes	419 (26.1)	187 (39.6)	219 (21.4)	13 (11.6)	
Medication use (*n*, %)					< 0.001
No	1276 (79.5)	296 (62.7)	869 (85.0)	111 (99.1)	
Yes	330 (20.5)	176 (37.3)	153 (15.0)	1 (0.9)	

### 3.2 Association Between LE8 and Mortality

During a median follow‐up period of 103 months, 282 participants died, of which 66 deaths were attributed to CVD. Prior to model construction, multicollinearity analysis revealed that all candidate predictors, including the LE8 score, exhibited adjusted VIF values ranging from 1.02 to 1.14 (Figure S1), well below the predefined threshold of 5. This confirms the absence of significant multicollinearity and over‐adjustment bias in our subsequent multivariable and machine learning analyses.

**Figure 3:**
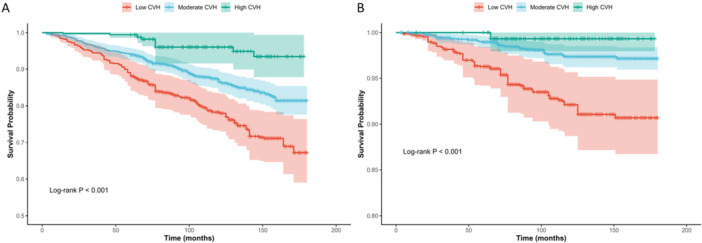
Unadjusted Kaplan‐Meier survival curves for (A) all‐cause mortality, and (B) cardiovascular disease (CVD) mortality among patients with sleep disorders, stratified by Life's Essential 8 (LE8) cardiovascular health (CVH) categories. The categories were defined as Low CVH (0–49), Moderate CVH (50–79), and High CVH (80–100). Differences in survival probabilities across the three strata were evaluated using the log‐rank test, demonstrating that higher LE8 scores are associated with significantly higher survival probabilities.

The Kaplan‐Meier survival curves (Figure 3) revealed a profound, dose‐dependent relationship between LE8 categories and mortality outcomes. Participants in the High CVH group demonstrated a remarkably superior survival probability throughout the follow‐up period compared to those in the Moderate and Low CVH groups (Log‐rank *p* < 0.001).

The weighted Cox regression analyses (Table 2) confirmed these findings. When analyzed as a continuous variable, a higher LE8 score was consistently associated with a reduced risk of mortality. In the fully adjusted model (Model 3), each 10‐point increment in the LE8 score was independently associated with a 15% reduction in all‐cause mortality risk (HR = 0.85, 95% CI, 0.73–0.99, *p* = 0.033) and a 28% reduction in CVD mortality risk (HR = 0.72, 95% CI, 0.56–0.93, *p* = 0.010). Similarly, when categorized by AHA criteria, a significant dose‐response gradient was observed across the LE8 categories for both all‐cause mortality (*p* for trend = 0.028) and CVD mortality (*p* for trend = 0.013).

**Table 2:** Association of LE8 scores with all‐cause and CVD mortality.

	Model 1		Model 2		Model 3	
	HR (95% CI)	*p*	HR (95% CI)	*p*	HR (95% CI)	*p*
**All‐cause mortality**						
LE8 score (per 10 points increase)	0.78 (0.70~0.88)	< 0.001	0.81 (0.70~0.93)	0.003	0.85 (0.73~0.99)	0.033
Categorical LE8 levels						
Low CVH (0–49)	1.00 (Reference)		1.00 (Reference)		1.00 (Reference)	
Moderate CVH (50–79)	0.54 (0.39~0.76)	< 0.001	0.62 (0.42~0.91)	0.016	0.71 (0.48~1.05)	0.094
High CVH (80–100)	0.18 (0.06~0.50)	0.001	0.29 (0.10~0.82)	0.020	0.35 (0.12~1.00)	0.050
*p* for trend	< 0.001		0.003		0.028	
**CVD mortality**						
LE8 score (per 10 points increase)	0.67 (0.56~0.81)	< 0.001	0.67 (0.52~0.85)	0.001	0.72 (0.56~0.93)	0.010
Categorical LE8 levels						
Low CVH (0–49)	1.00 (Reference)		1.00 (Reference)		1.00 (Reference)	
Moderate CVH (50–79)	0.30 (0.16~0.54)	< 0.001	0.35 (0.17~0.69)	0.003	0.43 (0.21~0.84)	0.014
High CVH (80–100)	0.08 (0.01~0.67)	0.019	0.14 (0.01~1.21)	0.074	0.20 (0.02~1.67)	0.137
*p* for trend	< 0.001		0.002		0.013	

Model 1: unadjusted model.

Model 2: adjusted for age, race, gender, education, marital status and poverty.

Model 3: fully adjusted for age, race, gender, education, marital status, poverty, alcohol consumption, baseline CVD status, depressive state and baseline medication use.

### 3.3 Dose‐Response Relationship

**Figure 4:**
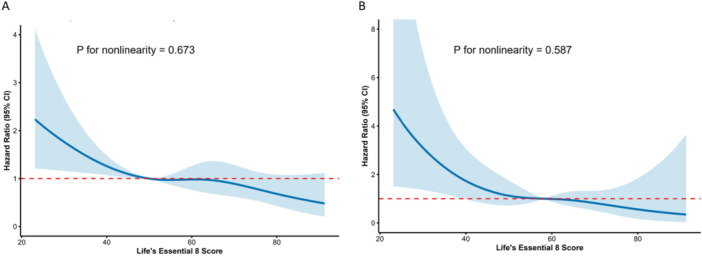
Restricted cubic spline (RCS) curves illustrating the dose‐response relationships between continuous Life's Essential 8 (LE8) scores and the risk of (A) all‐cause mortality, and (B) cardiovascular disease (CVD) mortality. The solid blue lines represent the estimated hazard ratios (HRs), and the shaded areas represent the 95% confidence intervals (CIs). The reference value (Hazard Ratio = 1.0) is indicated by the dashed red line. Both analyses were based on the fully adjusted Cox proportional hazards model (Model 3), which adjusted for age, gender, race, education, marital status, poverty‐to‐income ratio, alcohol consumption, baseline CVD status, depressive state, and baseline medication use.

The fully adjusted RCS analysis (Figure 4) suggested a near‐linear and inverse association between LE8 scores and the risk of all‐cause mortality (*p* for non‐linearity = 0.673) and CVD mortality (*p* for non‐linearity = 0.587), confirming that higher LE8 scores linearly confer a protective effect against mortality without any apparent threshold.

### 3.4 Subgroup Analysis

**Figure 5:**
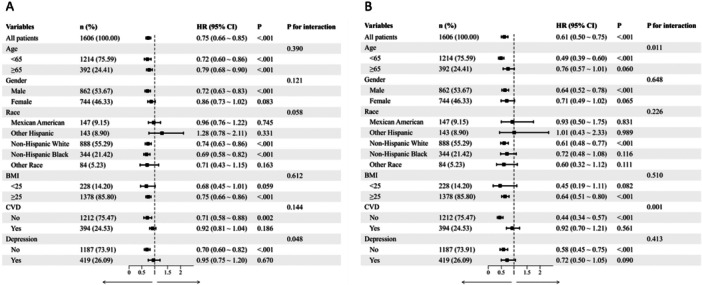
Forest plots of subgroup analyses evaluating the association between continuous Life's Essential 8 (LE8) scores and the risk of (A) all‐cause mortality, and (B) cardiovascular disease (CVD) mortality. Hazard ratios (HRs) and 95% confidence intervals (CIs) were calculated per 10‐point increment in the LE8 score. *p*‐values for interaction denote the significance of effect modification by each stratifying variable. The analyses were adjusted for age and gender.

Subgroup analyses were performed to evaluate the consistency of the prognostic value of the LE8 score across various clinical and demographic strata (Figure 5). Overall, the protective effect of higher LE8 scores against mortality remained robust across most subgroups. For all‐cause mortality (Figure 5A), a statistically significant interaction was observed with depressive state (*p* for interaction = 0.048). For CVD mortality (Figure 5B), significant effect modifications were identified for age (*p* for interaction = 0.011) and baseline CVD status (*p* for interaction = 0.001). The protective effect of the LE8 score against CVD mortality was remarkably stronger in younger individuals (HR = 0.49) and in patients without baseline CVD (HR = 0.44).

### 3.5 Establishment and Validation of the ML Based Prediction Model

**Figure 6:**
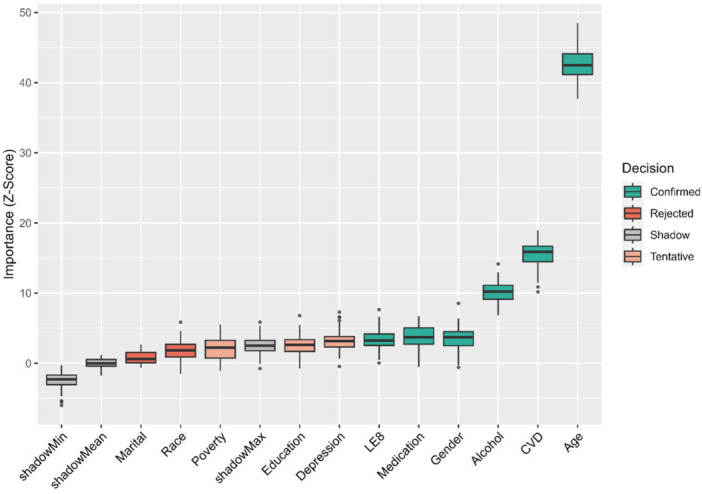
Feature selection for predicting all‐cause mortality based on the Boruta algorithm. The algorithm compared the importance of original clinical attributes against randomly shuffled “shadow” features. Green boxplots represented confirmed variables with significant predictive importance (*Z*‐scores significantly higher than the maximum *Z*‐score of shadow features), which were subsequently included in the machine learning models. Red boxplots indicate rejected features that lack predictive importance, and gray boxplots represent the shadow features used as a baseline threshold.

Feature selection was robustly performed using the Boruta algorithm prior to the development of ML models. As illustrated by Figure 6, variables confirmed as highly significant predictors (green boxplots) were subsequently utilized to develop the ML models.

**Figure 7:**
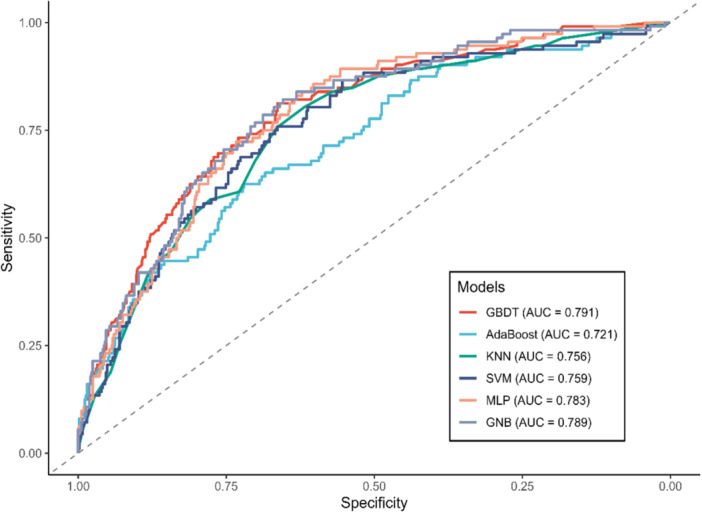
Receiver Operating Characteristic (ROC) curves comparing the predictive performance of six machine learning (ML) models for all‐cause mortality among patients with sleep disorders. The curves and corresponding Area Under the Curve (AUC) values reflected the models’ discriminative abilities when evaluated blindly on the testing set. AdaBoost, adaptive boosting; GBDT, gradient boosting decision tree; GNB, Gaussian naive bayes; KNN, K‐nearest neighbors; MLP, multi‐layer perceptron; SVM, support vector machine.

Following rigorous internal validation using fivefold cross validation, all six ML algorithms demonstrated acceptable to good discriminative abilities (Figure 7). The GBDT model achieved the highest overall discriminative ability with an AUC of 0.791, followed closely by the GNB model (AUC = 0.789). The smooth ROC curves of GBDT at high specificity thresholds (> 0.75) indicated its robust capacity to identify high‐risk individuals without excessively generating false‐positive alerts. The Table S2 presented the comprehensive performance metrics of the six models evaluated on the independent testing set. The GBDT achieved the highest *F*1‐score of 0.483, indicating an optimal balance between sensitivity and precision.

The calibration curves (Figure S2) illustrate the agreement between predicted probabilities and observed all‐cause mortality proportions across the six ML models. Notably, the GBDT model demonstrated the most robust calibration among all candidates, maintaining the closest proximity to the ideal reference line across the widest range of predicted probabilities. Furthermore, the DCA (Figure S3) indicated that the GBDT and GNB models provided the most substantial net clinical benefit across the clinically relevant threshold range (approximately 0% to 40%).

### 3.6 Sensitivity Analysis

The robustness of our findings was confirmed through three sensitivity analyses. Table S3 showed the association of LE8 scores with mortality risk after excluding patients who died within the first 2 years of follow‐up. Despite the anticipated loss of statistical power yielding borderline *p*‐values, the HRs remained virtually identical to the primary analysis (e.g., continuous HR for CVD mortality = 0.72, *p* = 0.028), confirming long‐term protective effects. Table S4 showed the association of tertiles of LE8 scores with mortality risk, which showed persisted dose‐response gradient, with the highest tertile exhibiting reduced mortality risks. The regression results after multiple imputation of missing data were showed in Table S5. Remarkably, with the increased statistical power, the protective associations became even more significant for both all‐cause mortality (continuous HR = 0.86, *p* = 0.002) and CVD mortality (continuous HR = 0.77, *p* = 0.009), unequivocally validating our primary findings.

## 4 Discussion

To the best of our knowledge, this is the first prospective cohort study to investigate the predictive significance of the LE8 score regarding mortality risk among individuals with sleep disorders. Our analysis revealed a robust dose‐dependent inverse association between higher CVH levels and significantly reduced risk of both all‐cause and CVD mortality in this demographic. Specifically, each 10‐point increment in the LE8 score reduced the risk of all‐cause mortality by 15% and the risk of CVD mortality by 28%, respectively. The absence of a non‐linear threshold (*p* for non‐linearity > 0.05) conveyed that CVH optimization operated on a continuum for the management of patients with sleep disorders. Furthermore, the integration of LE8 scores into modern ML algorithms, particularly the GBDT model, yielded exceptional predictive performance for all‐cause mortality. These findings imply that the LE8 metric is a highly valuable tool for risk stratification and emphasizes that comprehensive, progressive lifestyle and clinical modifications targeting all eight components of the LE8 framework should be aggressively pursued in this vulnerable population.

Our findings align with an expanding body of epidemiological research demonstrating the prognostic value of LE8 across various chronic diseases. For instance, prospective studies have linked elevated LE8 scores to decreased mortality in populations with type 2 diabetes [24] and insulin resistances [25]. In these populations, the protective effects of LE8 are heavily mediated by reductions in systemic inflammation and vascular aging. This pathophysiological mechanism likely extends to individuals with sleep disorders [26]. Sleep disturbances, such as shortened sleep duration and poor sleep quality, are established risk factors for atherosclerosis, myocardial infarction [27, 28], and increased carotid intima‐media thickness [29]. The mechanisms bridging sleep disorders and cardiovascular events are heavily rooted in inflammation. Sleep deprivation triggers the secretion of pro‐inflammatory cytokines (e.g., interleukin‐1 and TNF‐alpha), activates C‐reactive protein, inhibits endothelium‐dependent vasodilation, and drives sympathetic nervous system overactivity [30, 31, 32, 33, 34, 35]. Because poor LE8 scores reflect compounding behavioral risks (like poor diet and physical inactivity) that also drive oxidative stress, optimizing the modifiable factors within the LE8 framework can synergistically mitigate the inflammatory cascade inherent to sleep disorders [36, 37].

Our categorical analysis intriguingly demonstrated a robust dose‐response gradient (*p* for trend < 0.05), despite the non‐significant *p*‐value observed in the High CVH versus Low CVH pairwise comparison. The non‐significant pairwise *p*‐value and excessively wide 95% CI in the High CVH group is a classic statistical artifact of power depletion, driven by the exceptionally low event rate among individuals with optimal CVH. While this exceptionally low event rate biologically underscores the potent protective effect of maintaining ideal CVH metrics, it inherently restricts statistical precision and limits the reliability and interpretability of the point estimate for the High CVH category. To mitigate this analytical constraint, we relied on the continuous LE8 score analysis and the test for trend across ordinal categories, both of which maximize statistical power by utilizing the entire data distribution.

Interestingly, our subgroup analyses identified a significant interaction between LE8 scores and pre‐existing CVD. The cardioprotective efficacy of higher LE8 scores was substantially blunted in patients who already had CVD at baseline. A plausible explanation is that individuals with both sleep disorders and established CVD may possess advanced structural cardiac alterations (such as severe atherosclerosis or myocardial fibrosis) that overshadow the short‐term benefits of modifiable lifestyle factors [38]. Additionally, these patients are likely receiving aggressive pharmacological interventions, which inherently modifies their blood pressure, lipid, and glucose metrics, thereby diluting the natural predictive association of the LE8 score. We also observed an age interaction, wherein the cardiovascular benefits of high LE8 scores were less pronounced in older adults. Aging is associated with diminished physiological responsiveness to health‐promoting behaviors, characterized by reduced insulin sensitivity and impaired endothelial repair [39]. Age‐related effect modification highlights the necessity of early LE8 intervention to maximize cardiovascular benefits before physiological responsiveness wanes with age [40].

By leveraging machine learning, we successfully developed a highly reliable and clinically achievable predictive model (GBDT AUC = 0.791), which struck the most clinically viable balance, achieving a positive predictive value (PPV) of 0.448 alongside a sensitivity of 0.527. Compared to traditional risk models, this ML‐based approach elegantly captures the complex, non‐linear interactions among variables. For clinicians, this represents a refined, personalized tool for the early identification of high‐risk patients within the sleep disorder population, facilitating timely and targeted interventions.

The findings of this study carry profound clinical implications for the comprehensive management of patients with sleep disorders. Traditionally, the clinical focus for this demographic has been heavily skewed towards the direct alleviation of sleep‐related symptoms, often underappreciating the systemic cardiovascular sequelae. Our data strongly advocate for a paradigm shift toward an integrated, holistic care model utilizing the LE8 framework. Because sleep disorders are intrinsically linked to neuroendocrine dysregulation, heightened sympathetic tone, and metabolic disturbances, all of which potently accelerate cardiovascular events, the LE8 score serves as a critical, quantifiable bridge between sleep medicine and preventive cardiology. We propose that the LE8 metric should be systematically incorporated into the routine clinical screening of all patients presenting with sleep disturbances. Operationally, this could involve the automated calculation of LE8 scores within electronic health records (EHRs) during primary care or sleep clinic visits, utilizing readily available data (e.g., BMI, blood pressure, fasting glucose, and self‐reported behavioral metrics).

This study has several limitations. First, the diagnosis of sleep disorders was ascertained through self‐reported questionnaires rather than objective clinical evaluations, such as polysomnography or actigraphy. This reliance on self‐reporting introduced a significant potential for misclassification bias. Participants might underreport undiagnosed conditions (e.g., asymptomatic obstructive sleep apnea) or, conversely, conflate transient sleep disturbances with chronic clinical disorders. Such misclassification could blur the true associations between LE8 scores and mortality, potentially underestimating the true magnitude of the observed protective effects. Similarly, the dietary, physical activity, and smoking components of the LE8 score, were also derived from self‐reported data, which are inherently susceptible to recall bias. Second, although specific sleep disorder subtypes were recorded in the earliest survey cycles (2005–2008), this detailed information was unavailable for the majority of the study period (2009–2014). This data inconsistency, coupled with the lack of objective polysomnography data, precluded adequately powered subgroup analyses based on specific sleep disorder phenotypes. Third, while our ML models (particularly GBDT) demonstrated excellent predictive performance, they possess inherent methodological limitations. Advanced ML algorithms frequently operate as “black boxes”, lacking the straightforward interpretability and easily quantifiable HRs of traditional Cox regression. Finally, our ML models lack external validation. Although we employed a fivefold cross validation strategy within the training set to actively mitigate internal overfitting, the models were derived and trained exclusively on a single, historical national database. Consequently, there is a persistent risk that the algorithms may have overfitted to the specific demographic distributions, socioeconomic conditions, and healthcare characteristics unique to this cohort. The generalizability of these predictive models to more populations remains unverified. Future prospective studies incorporating independent, external validation cohorts are imperative to confirm the reproducibility and clinical reliability of these ML algorithms before they can be broadly implemented.

## 5 Conclusion

This study demonstrates a significant, independent, and inverse association between the LE8 score and both all‐cause and CVD mortality in adults with sleep disorders. ML‐based predictive modeling incorporating LE8 achieved excellent predictive performance. These findings underscore the critical clinical utility of the LE8 framework as an early risk stratification tool and advocate for aggressive, comprehensive CVH management in patients suffering from sleep disorders.

## Author Contributions

**Jia Wei:** conceptualization, methodology, formal analysis, writing – original draft, writing – review and editing. **Tengfei Ji:** conceptualization, methodology, formal analysis, writing – original draft, writing – review and editing. **Yide Yuan:** visualization, investigation. **Su Liu:** visualization, investigation, writing – review and editing. **Qing Yan:** writing – review and editing. **YuYang Zhao:** writing – review and editing. **Lan Yang:** writing – review and editing. **Jiahong Xue:** Funding acquisition.

## Ethics Statement

The National Center for Health Statistics (NCHS) and the Centers for Disease Control and Prevention (CDC) carry out NHANES. The NHANES study protocol was evaluated and approved by the NCHS Research Ethics Review Committee.

## Consent

Written informed permission was signed by each participant.

## Conflicts of Interest

The authors declare no conflicts of interest.

## Supporting information

Supporting File

## Data Availability

The data that support the findings of this study are available in NHANES at https://wwwn.cdc.gov/nchs/nhanes/default.aspx. These data were derived from the following resources available in the public domain: 2005–2006, https://wwwn.cdc.gov/nchs/nhanes/continuousnhanes/default.aspx?BeginYear=2005
2007–2008, https://wwwn.cdc.gov/nchs/nhanes/continuousnhanes/default.aspx?BeginYear=2007
2009–2010, https://wwwn.cdc.gov/nchs/nhanes/continuousnhanes/default.aspx?BeginYear=2009
2011–2012, https://wwwn.cdc.gov/nchs/nhanes/continuousnhanes/default.aspx?BeginYear=2011
2013–2014, https://wwwn.cdc.gov/nchs/nhanes/continuousnhanes/default.aspx?BeginYear=2013 Data from the National Health and Nutrition Examination Survey (NHANES) 2005–2014 are publicly available online (https://wwwn.cdc.gov/nchs/nhanes/Default.aspx).
