# Supplementary material for: The Correlations and Predictive Capabilities of the “Life's Essential 8” With Respect to All‐Cause Mortality and Cardiovascular Disease Mortality Risks in Individuals Experiencing Sleep Disorders: A Prospective Cohort Study From the NHANES (2005–2014)
Source: Clin Cardiol. 2026 Apr 27;49(5):e70336. doi: 10.1002/clc.70336 (PMC13112593; doi:10.1002/clc.70336)
Supplement: Supplementary file 1 — Supporting File [file CLC-49-e70336-s001.docx]

**Supplemental Material**

Supplementary Table S1. Definition and scoring approach for the Life’s Essential 8 (LE8) score

| **LE8 component** | **Method of measurement** | **Criteria** | **Points** |
| --- | --- | --- | --- |
| Diet | Quantiles of HEI-2015 (population) | 1st–24th | 0 |
|  |  | 25th–49th | 25 |
|  |  | 50th–74th | 50 |
|  |  | 75th–94th | 80 |
|  |  | ≥95th | 100 |
| Physical activity | Self-reported minutes of moderate or vigorous activities per week | 0 | 0 |
|  |  | 1–29 | 20 |
|  |  | 30–59 | 40 |
|  |  | 60–89 | 60 |
|  |  | 90–119 | 80 |
|  |  | 120–149 | 90 |
|  |  | ≥150 | 100 |
| Nicotine exposure | Self-reported use of cigarettes or inhaled NDS | Current smoker | 0 |
|  |  | Former smoker, quit <1 y, living with indoor smoker | 5 |
|  |  | Former smoker, quit <1 y, or using inhaled NDS | 25 |
|  |  | Former smoker, quit 1–<5 y, living with indoor smoker | 30 |
|  |  | Former smoker, quit 1–<5 y | 50 |
|  |  | Former smoker, quit ≥5 y, living with indoor smoker | 55 |
|  |  | Former smoker, quit ≥5 y | 75 |
|  |  | Never smoker, living with indoor smoker | 80 |
|  |  | Never smoker | 100 |
| Sleep health | Self-reported average hours of sleep per night | <4 h | 0 |
|  |  | 4–<5 h | 20 |
|  |  | 5–<6 or ≥10 h | 40 |
|  |  | 6–<7 h | 70 |
|  |  | 9–<10 h | 90 |
|  |  | 7–<9 h | 100 |
| BMI | Body weight (kilograms) divided by height squared | ≥40.0 | 0 |
|  |  | 35.0–39.9 | 15 |
|  |  | 30.0–34.9 | 30 |
|  |  | 25.0–29.9 | 70 |
|  |  | <25 | 100 |
| Blood lipids | Plasma total and HDL cholesterol with calculation of non-HDL cholesterol | ≥220 or 190–219 and using medication | 0 |
|  |  | 190–219 or160–189 and using medication | 20 |
|  |  | 160–189 or 130–159 and using medication | 40 |
|  |  | 130–159 | 60 |
|  |  | <130 and using medication | 80 |
|  |  | <130 | 100 |
| Blood glucose | HbA_1c_ (%) | Diabetes with HbA_1c_ ≥10.0 | 0 |
|  |  | Diabetes with HbA_1c_ 9.0–9.9 | 10 |
|  |  | Diabetes with HbA_1c_ 8.0–8.9 | 20 |
|  |  | Diabetes with HbA_1c_ 7.0–7.9 | 30 |
|  |  | Diabetes with HbA_1c_ <7.0 | 40 |
|  |  | No diabetes and HbA_1c_ 5.7–6.4 | 60 |
|  |  | No diabetes an HbA_1c_ <5.7 | 100 |
| Blood pressure | Systolic and diastolic BPs (mm Hg) | ≥160 or ≥100 | 0 |
|  |  | 140–159 or 90–99 and using medication | 5 |
|  |  | 140–159 or 90–99 (no medication) | 10 |
|  |  | 130–139 or 80–89 and using medication | 30 |
|  |  | 130–139 or 80–89 (no medication) | 50 |
|  |  | 120–129/<80 and using medication | 55 |
|  |  | 120–129/<80 | 75 |
|  |  | <120/<80 and using medication | 80 |
|  |  | <120/<80 | 100 |


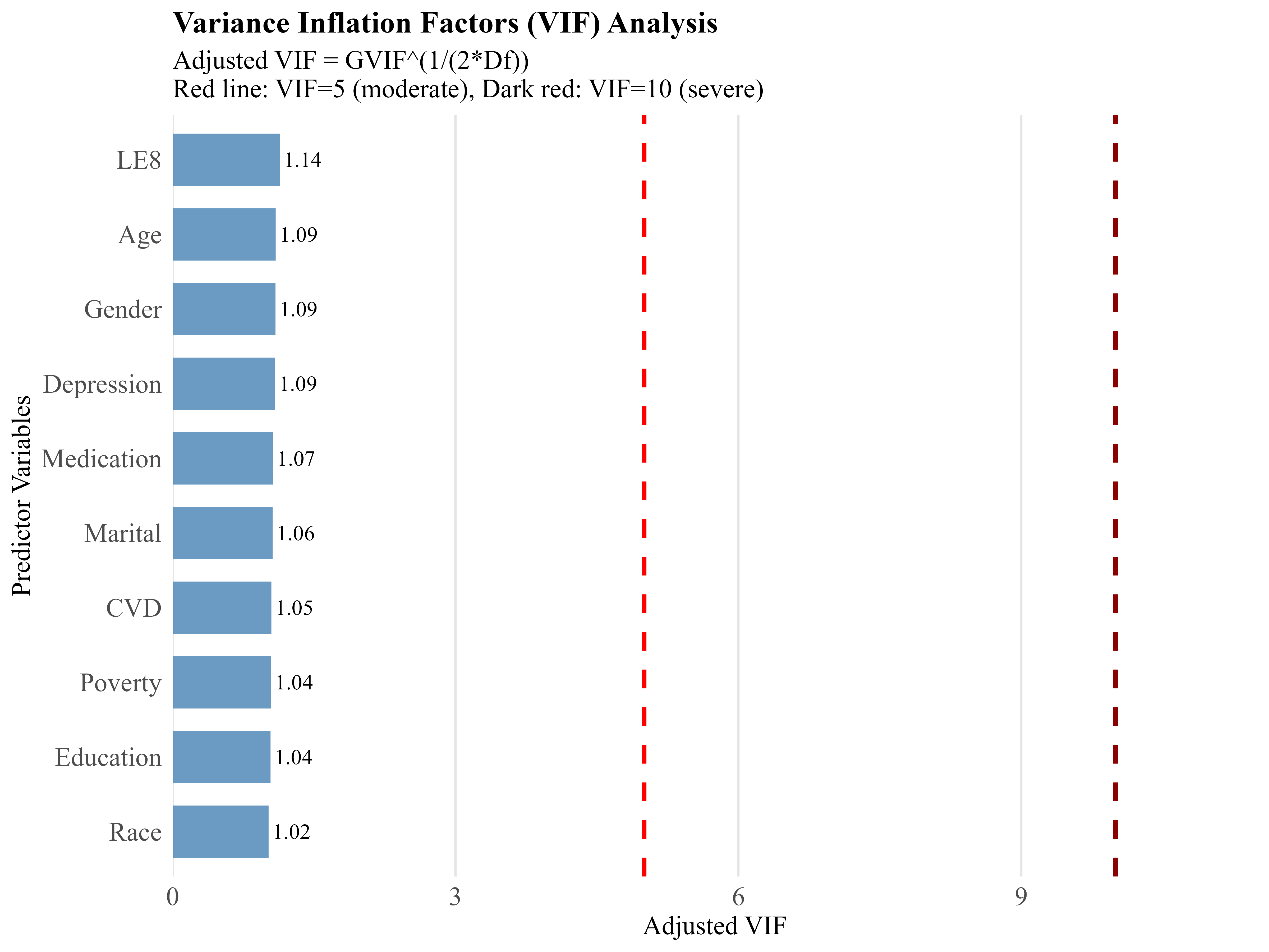


Supplementary Figure S1. Assessment of multicollinearity among the candidate predictor variables. The bar chart displays the adjusted Variance Inflation Factor (VIF) for each variable included in the fully adjusted multivariable models. The dashed red line indicates the predefined threshold for moderate collinearity (adjusted VIF = 5), and the dark red dashed line indicates severe collinearity (adjusted VIF = 10).

Supplementary Table S2. Comprehensive predictive performance metrics of the six machine learning algorithms evaluated on the independent testing set for all-cause mortality.

| **Model** | **AUC (SD)** | **Accuracy (SD)** | **Sensitivity (SD)** | **Positive predictive value (SD)** | **Negative predictive value (SD)** | **F1 (SD)** |
| --- | --- | --- | --- | --- | --- | --- |
| **GBDT** | 0.791 (0.023) | 0.804 (0.015) | 0.527 (0.048) | 0.448 (0.043) | 0.896 (0.013) | 0.483 (0.040) |
| **AdaBoost** | 0.722 (0.027) | 0.802 (0.015) | 0.374 (0.044) | 0.424 (0.049) | 0.871 (0.014) | 0.397 (0.041) |
| **KNN** | 0.756 (0.024) | 0.745 (0.017) | 0.590 (0.048) | 0.359 (0.035) | 0.900 (0.014) | 0.446 (0.036) |
| **SVM** | 0.760 (0.024) | 0.753 (0.017) | 0.581 (0.047) | 0.368 (0.036) | 0.899 (0.014) | 0.450 (0.036) |
| **MLP** | 0.784 (0.022) | 0.706 (0.018) | 0.733 (0.041) | 0.342 (0.030) | 0.925 (0.013) | 0.465 (0.033) |
| **GNB** | 0.789 (0.022) | 0.821 (0.014) | 0.312 (0.043) | 0.480 (0.055) | 0.864 (0.014) | 0.377 (0.044) |


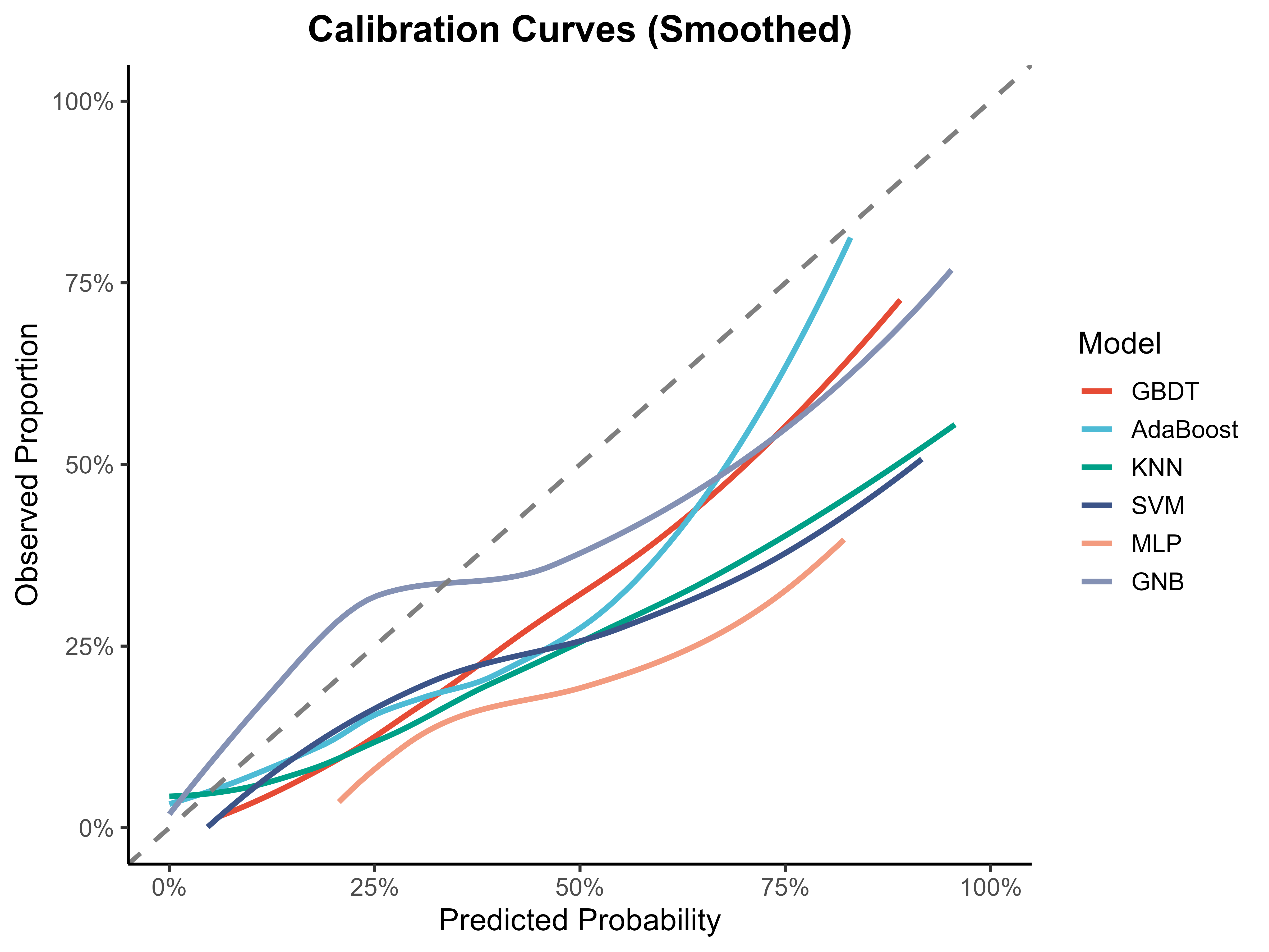


Supplementary Figure S2. Calibration curve evaluating the predictive accuracy of the six machine learning algorithms for all-cause mortality. The performance was evaluated on the independent testing set. The x-axis represents the predicted probability of mortality generated by the respective models, and the y-axis represents the actual observed proportion of mortality. The diagonal dashed grey line denotes the ideal nomogram (perfect calibration). Curves maintaining a closer proximity to this diagonal reference line indicate superior calibration and higher reliability of the predicted risk estimates.


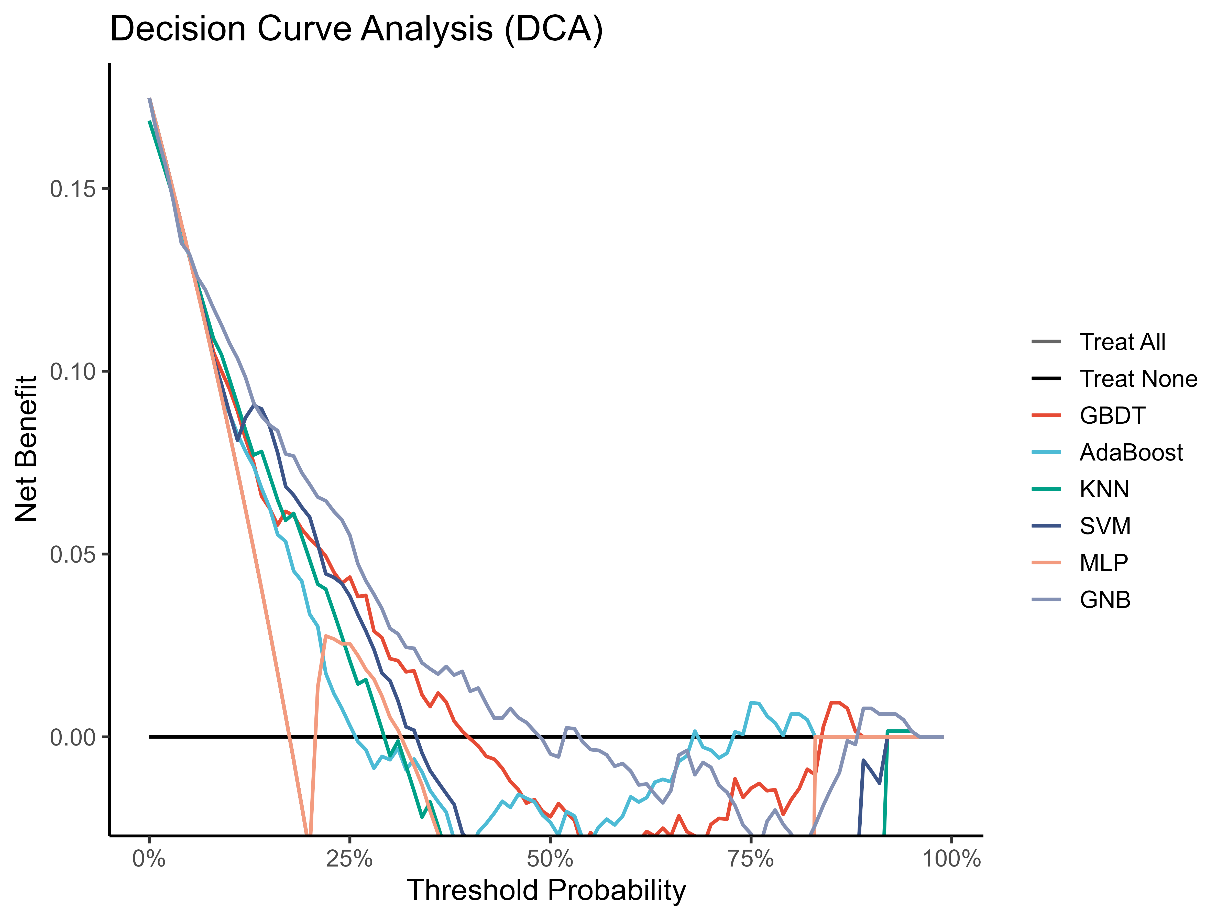


Supplementary Figure S3. Decision curve analysis (DCA) assessing the clinical utility of the six machine learning algorithms. The x-axis indicates the threshold probability (the point at which a patient would be classified as high-risk), and the y-axis indicates the net clinical benefit.

Supplementary Table S3. The association of LE8 scores with all-cause and CVD mortality after excluding samples with a follow-up period of fewer than two years

|  | **Model 1** | | **Model 2** | | **Model 3** | |
| --- | --- | --- | --- | --- | --- | --- |
|  | HR (95% CI) | *P* | HR (95% CI) | *P* | HR (95% CI) | *P* |
| **All-cause mortality** | | | | | | |
| LE8 score (Per 10 points increase) | 0.78 (0.68 ~ 0.90) | <0.001 | 0.80 (0.67 ~ 0.96) | 0.017 | 0.84 (0.70 ~ 1.02) | 0.083 |
| LE8 levels | | | | | | |
| Low CVH (0–49) | 1.00 (Reference) |  | 1.00 (Reference) |  | 1.00 (Reference) |  |
| Moderate CVH (50–79) | 0.53 (0.36 ~ 0.80) | 0.003 | 0.60 (0.36 ~ 0.99) | 0.047 | 0.69 (0.42 ~ 1.14) | 0.155 |
| High CVH (80–100) | 0.20 (0.07 ~ 0.60) | 0.004 | 0.31 (0.10 ~ 0.97) | 0.044 | 0.38 (0.12 ~ 1.17) | 0.093 |
| *P* for trend | <0.001 | | 0.019 | | 0.072 | |
| **CVD mortality** | | | | | | |
| LE8 score (Per 10 points increase) | 0.69 (0.56 ~ 0.85) | <0.001 | 0.68 (0.51 ~ 0.90) | 0.008 | 0.72 (0.54 ~ 0.96) | 0.028 |
| LE8 levels | | | | | | |
| Low CVH (0–49) | 1.00 (Reference) |  | 1.00 (Reference) |  | 1.00 (Reference) |  |
| Moderate CVH (50–79) | 0.30 (0.16 ~ 0.58) | <0.001 | 0.35 (0.16 ~ 0.75) | 0.007 | 0.41 (0.20 ~ 0.85) | 0.017 |
| High CVH (80–100) | 0.10 (0.01 ~ 0.79) | 0.029 | 0.15 (0.02 ~ 1.38) | 0.094 | 0.20 (0.02 ~ 1.66) | 0.136 |
| *P* for trend | <0.001 | | 0.007 | | 0.017 | |

Model 1: unadjusted model

Model 2: adjusted for age, race, gender, education, marital status and poverty

Model 3: fully adjusted for age, race, gender, education, marital status, poverty, alcohol consumption, CVD status, depressive state and baseline medication use

Supplementary Table S4. The association of LE8 scores with all-cause, and CVD mortality according to tertiles of LE8 scores

|  | **Model 1** | | **Model 2** | | **Model 3** | |
| --- | --- | --- | --- | --- | --- | --- |
|  | HR (95% CI) | *P* | HR (95% CI) | *P* | HR (95% CI) | *P* |
| **All-cause mortality** | | | | | | |
| LE8 levels | | | | | | |
| Tertile 1 | 1.00 (Reference) |  | 1.00 (Reference) |  | 1.00 (Reference) |  |
| Tertile 2 | 0.57 (0.40 ~ 0.82) | 0.002 | 0.65 (0.45 ~ 0.94) | 0.023 | 0.72 (0.49 ~ 1.08) | 0.117 |
| Tertile 3 | 0.46 (0.31 ~ 0.67) | <0.001 | 0.56 (0.37 ~ 0.86) | 0.009 | 0.68 (0.44 ~ 1.05) | 0.080 |
| *P* for trend | <0.001 | | 0.009 | | 0.080 | |
| **CVD mortality** | | | | | | |
| LE8 levels | | | | | | |
| Tertile 1 | 1.00 (Reference) |  | 1.00 (Reference) |  | 1.00 (Reference) |  |
| Tertile 2 | 0.31 (0.15 ~ 0.63) | 0.001 | 0.36 (0.18 ~ 0.74) | 0.005 | 0.43 (0.20 ~ 0.88) | 0.022 |
| Tertile 3 | 0.30 (0.16 ~ 0.56) | <0.001 | 0.40 (0.19 ~ 0.83) | 0.014 | 0.54 (0.26 ~ 1.10) | 0.093 |
| *P* for trend | <0.001 | | 0.015 | | 0.080 | |

Model 1: unadjusted model

Model 2: adjusted for age, race, gender, education, marital status and poverty

Model 3: fully adjusted for age, race, gender, education, marital status, poverty, alcohol consumption, CVD status, depressive state and baseline medication use

Supplementary Table S5. The association of LE8 scores with all-cause, and CVD mortality after performing multiple imputation for missing data

|  | **Model 1** | | **Model 2** | | **Model 3** | |
| --- | --- | --- | --- | --- | --- | --- |
|  | HR (95% CI) | *P* | HR (95% CI) | *P* | HR (95% CI) | *P* |
| **All-cause mortality** | | | | | | |
| LE8 score (Per 10 points increase) | 0.79 (0.72 ~ 0.85) | <0.001 | 0.81 (0.74 ~ 0.90) | <0.001 | 0.86 (0.78 ~ 0.94) | 0.002 |
| LE8 levels | | | | | | |
| Low CVH (0–49) | 1.00 (Reference) |  | 1.00 (Reference) |  | 1.00 (Reference) |  |
| Moderate CVH (50–79) | 0.60 (0.46 ~ 0.80) | <0.001 | 0.70 (0.52 ~ 0.91) | 0.009 | 0.78 (0.58 ~ 1.07) | 0.130 |
| High CVH (80–100) | 0.18 (0.04 ~ 0.73) | 0.017 | 0.29 (0.07 ~ 1.15) | 0.079 | 0.35 (0.09 ~ 1.32) | 0.122 |
| *P* for trend | <0.001 | | <0.001 | | 0.018 | |
| **CVD mortality** | | | | | | |
| LE8 score (Per 10 points increase) | 0.68 (0.60 ~ 0.78) | <0.001 | 0.72 (0.60 ~ 0.86) | <0.001 | 0.77 (0.63 ~ 0.94) | 0.009 |
| LE8 levels | | | | | | |
| Low CVH (0–49) | 1.00 (Reference) |  | 1.00 (Reference) |  | 1.00 (Reference) |  |
| Moderate CVH (50–79) | 0.38 (0.25 ~ 0.58) | <0.001 | 0.48 (0.31 ~ 0.73) | <0.001 | 0.57 (0.35 ~ 0.92) | 0.021 |
| High CVH (80–100) | 0.07 (0.01 ~ 1.38) | 0.081 | 0.14 (0.01 ~ 2.12) | 0.158 | 0.20 (0.01 ~ 2.73) | 0.226 |
| *P* for trend | <0.001 | | <0.001 | | 0.007 | |

Model 1: unadjusted model

Model 2: adjusted for age, race, gender, education, marital status and poverty

Model 3: fully adjusted for age, race, gender, education, marital status, poverty, alcohol consumption, CVD status, depressive state and baseline medication use
